# Magnetic resonance imaging for characterization of hepatocellular carcinoma metabolism

**DOI:** 10.3389/fphys.2022.1056511

**Published:** 2022-12-15

**Authors:** Lixia Wang, Ju Dong Yang, Charles C. Yoo, Keane K. Y. Lai, Jonathan Braun, Dermot P. B. McGovern, Yibin Xie, Stephen J. Pandol, Shelly C. Lu, Debiao Li

**Affiliations:** ^1^ Biomedical Imaging Research Institute, Cedars-Sinai Medical Center, Los Angeles, CA, United States; ^2^ Samuel Oschin Comprehensive Cancer Institute, Cedars-Sinai Medical Center, Los Angeles, CA, United States; ^3^ Karsh Division of Gastroenterology and Hepatology, Cedars-Sinai Medical Center, Los Angeles, CA, United States; ^4^ Comprehensive Transplant Center, Cedars-Sinai Medical Center, Los Angeles, CA, United States; ^5^ Office of the Medical Director 1st MRI, Los Angeles, CA, United States; ^6^ Department of Molecular Medicine, Beckman Research Institute of City of Hope and City of Hope Comprehensive Cancer Center, Duarte, CA, United States; ^7^ F. Widjaja Inflammatory Bowel Disease Institute, Division of Digestive and Liver Diseases, Department of Medicine, Cedars-Sinai Medical Center, Los Angeles, CA, United States; ^8^ Division of Digestive and Liver Diseases, Cedars-Sinai Medical Center, Los Angeles, CA, United States; ^9^ Department of Bioengineering, University of California, Los Angeles, CA, United States

**Keywords:** hepatocellular carcinoma, metabolism, tumor micro environment, hypoxia, magnetic resonance imaging, positron emission tomography/MR

## Abstract

With a better understanding of the pathophysiological and metabolic changes in hepatocellular carcinoma (HCC), multiparametric and novel functional magnetic resonance (MR) and positron emission tomography (PET) techniques have received wide interest and are increasingly being applied in preclinical and clinical research. These techniques not only allow for non-invasive detection of structural, functional, and metabolic changes in malignant tumor cells but also characterize the tumor microenvironment (TME) and the interactions of malignant tumor cells with the TME, which has hypoxia and low pH, resulting from the Warburg effect and accumulation of metabolites produced by tumor cells and other cellular components. The heterogeneity and complexity of the TME require a combination of images with various parameters and modalities to characterize tumors and guide therapy. This review focuses on the value of multiparametric magnetic resonance imaging and PET/MR in evaluating the structural and functional changes of HCC and in detecting metabolites formed owing to HCC and the TME.

## Introduction

Hepatocellular carcinoma (HCC) is the third leading cause of cancer-related death and the sixth most common carcinoma (Sung et al., 2021). In adults, HCC is usually a malignant tumor and often develops from chronic liver disease (Bruix et al., 2004). Chronic hepatitis, cirrhosis, heavy drinking, and other hereditary or metabolic liver disorders are risk factors for HCC. The characteristics of HCC include cell proliferation, anti-apoptosis, angiogenesis, tumor invasion, and metastasis. To cope with the progression of tumor proliferation, there is a high requirement for macromolecular biosynthesis, structural change and destruction, energy supply, and a suitable tumor microenvironment (TME), particularly for energy metabolism. In normal cells, glucose catabolism is achieved through oxidative phosphorylation in the mitochondria, while malignant cells generate energy *via* aerobic glycolysis, which converts glucose into lactate even under abundant oxygen supply; this phenomenon was first described by Warburg (O 1925). Nutrients for tumor growth and invasion are mainly provided by glucose metabolism, oxidative phosphorylation, tricarboxylic acid cycling, and lipid metabolism.

Tumor growth, invasion, and metastasis also depend on the TME. The TME of HCC has high heterogeneity and is composed of both noncellular and cellular components. Within the tumor and surrounding tissues, abundant immune inflammatory cells accumulate owing to tumor-associated inflammation. The cellular components are HCC-associated fibroblasts, immune cells, and other cells. Extracellular proteins, enzymes, inflammatory cytokines, and other molecules constitute the tumor stroma. These components constitute the surrounding environment of cancer cells and play an important role in the metabolic progress of cancer cells and the interaction between cancer cells and the surrounding environment (Yang et al., 2011; Hernandez-Gea et al., 2013; Ramamonjisoa and Ackerstaff 2017). The characteristics of the TME are hypoxia, low pH, and many metabolites that are produced by tumor cells and other cellular components. Owing to aerobic glycolysis and the hypoxic TME, malignant cells prefer to convert glucose to lactic acid. The dissociated components of lactic acid render the extracellular tumor pH (pHe) more acidic.

Because of the heterogeneity and complexity of the TME in HCC, multimodality magnetic resonance imaging (MRI) or multiparametric MRI are designed to utilize the advantages of techniques while reducing their disadvantages, which can provide detailed pathophysiological information and reflect changes in tumor intra-structure, metabolism, hypoxia, acidic microenvironment, and lack of hepatocyte-specific contrast agent uptake.

## Magnetic resonance (MR) techniques in clinical practice for HCC metabolism

### Diffusion-weighted imaging (DWI) and intravoxel incoherent motion (IVIM)

DWI is one of the most promising non-contrast MRI sequences that allows for tumor detection and differentiation. The apparent diffusion coefficient (ADC) values derived from DWI can quantitatively measure water diffusion mobility and indicate tissue characteristics, such as cellular density and extracellular extravascular volume. HCC shows hyperintensity on DWI with a high b value (500–1,000 s/mm^2^) and can be used to differentiate it from dysplastic nodules (DNs) (Vandecaveye et al., 2009). Using the minimum ADC value of histogram-derived parameters, poorly differentiated HCC can be distinguished from well-differentiated and moderately differentiated HCCs, achieving a high sensitivity of 100% (Moriya et al., 2017). Compared with other parameters, the minimum ADC achieved the highest area under the curve (AUC) of 0.763 in discriminating well to moderately differentiated HCC from poorly differentiated HCC, which reflects the heterogeneous and hypercellular component within the tumor (Xu et al., 2019). Cellularity increases with the increase in tumor grade, which restricts molecular diffusion of water.

Another study found that elevated serum alpha-fetoprotein (AFP) and lower 75th percentile ADC values were independent risk factors with sensitivity (79%) and specificity (79.1%) for glypican-3 (GPC-3)-positive HCC. GPC-3 overexpression in HCC cells is involved in cell growth, migration, differentiation, and invasion. As an immune therapeutic target, it plays an important role in monoclonal antibody therapies. Combining AFP and lower 75th percentile ADC values can provide early prediction and effective treatment guidance (Zhao et al., 2021). Several researchers observed that the distributed diffusion coefficient derived from the stretched-exponential model and ADC were significantly lower in tumors with microvascular invasion than in those without microvascular invasion. These decreases were associated with microvascular invasion, cell proliferation, increased nuclear-to-cytoplasmic ratio, and a complicated tumor microenvironment, which further restricts the diffusion of water molecules (Li et al., 2022).

**FIGURE 1:**
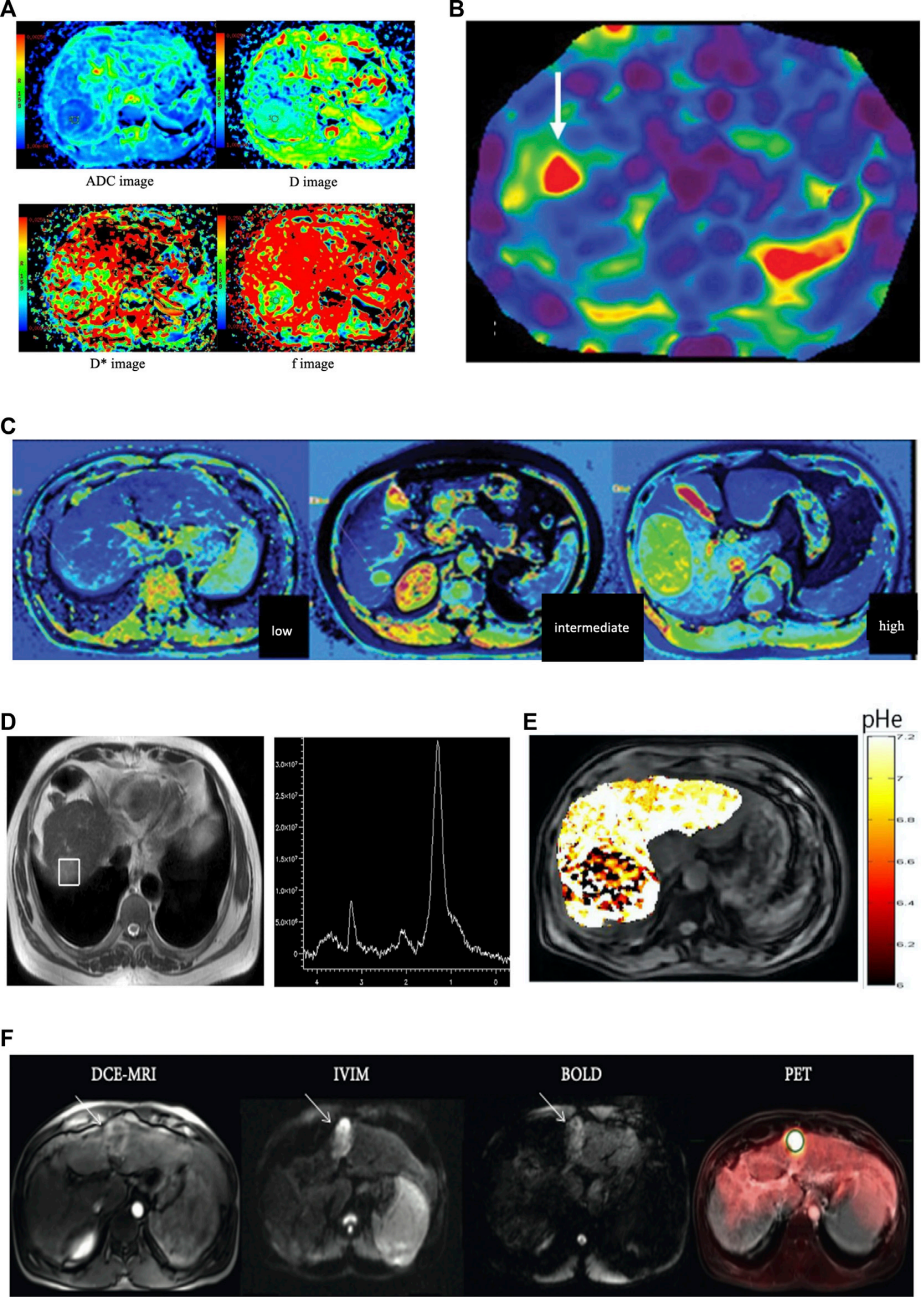
**(A)** Imaging of a 50-year-old female patient with high grade hepatocellular carcinoma in the right lobe. Diffusion-weighted imaging (DWI) derived apparent diffusion coefficient map (ADC), true molecular-diffusion coefficient map (D), perfusion related diffusion coefficient map (D*), and the perfusion fraction map (f). The measured values were 1.56 × 10^–3^ mm^2^/s, 1.04 × 10^–3^ mm^2^/s, 15.50 × 10^–3^ mm^2^/s, and 0.29, respectively (the figure was reprinted with minor revision from Springer Nature (Zhou et al., 2021). **(B)** MRE of a 63-year-old patient with HCC concomitant with chronic hepatitis. The mean stiffness of the hepatocellular carcinoma was 8.2 kPa, and the background hepatic stiffness of only 2.9 kPa that was suggestive of a non-cirrhotic liver parenchyma (the figure was reprinted from Xia and He Publishing Inc. (Navin and Venkatesh 2019)). **(C)** T1-mapping of low grade, intermediate grade, and high-gradeHCC (left to right). The T1 values were 892 ms, 1,696 ms,and 2,134 ms, respectively (the figure was reprinted with minor revision from Xia and He Publishing Inc. (Navin and Venkatesh 2019). **(D)** Localized magnetic resonance images and 1H magnetic resonance spectra in hepatocellular carcinoma. Localized magnetic resonance image shows the location of the voxel of the tumor. A high choline-containing compound peak at 3.2 ppm was detected in the tumor in 1H MRS (the figure was reprinted from Springer Nature (Zhang et al., 2016)). **(E)** pHe map of HCC. The CEST signal for the tumor area differed from the corresponding color of the adjacent normal liver tissue, with a lower pHe value in the tumor area than in the normal tissue (the figure was reprinted from frontiers Media S.A (Tang et al., 2020)). **(F)** A 51-year-old male patient with cirrhosis secondary to chronic HCV and HCC. DCE-MRI, IVIM (*b* = 400), and BOLD (TE = 30 ms, pre-O_2_) images and PET overlay on anatomical T_2_-weighted image demonstrating 3.8 cm HCC in the left liver lobe (white arrows). The HCC lesion showed avid FDG uptake (SUV_mean_ 6.06 and SUV_max_ 7.80) and relatively low perfusion/permeability as measured by DCE-MRI (the figure was reprinted from Hindawi publisher (Hectors, Wagner et al., 2018)).

IVIM-DWI combines low and high b values to estimate perfusion resulting from microvascular flow and water molecular diffusion effects resulting from cellular density, respectively. This may allow for the prediction of the degree of histological differentiation and assessment of radioembolization effects. The parameters derived from IVIM using the biexponential IVIM model included the pseudo-diffusion (perfusion-related) coefficient (D*), true diffusion coefficient (D), and perfusion fraction (F) (Figure 1A). Zhou (Zhou et al., 2021) analyzed 70 patients with HCC and found that the diagnostic sensitivities and specificities were 73.3% and 85.5% with ADC and 86.7% and 78.2% with D, respectively. The AUCs were 0.821 (cut-off value of ADC: 1.25 × 10^–3^ mm^2^/s) and 0.841 (cut-off value of D: 0.97 × 10^–3^ mm^2^/s) for distinguishing well-differentiated HCC from moderately differentiated and poorly differentiated HCCs, respectively. Hectors (Hectors et al., 2020) analyzed 25 HCCs and found significantly decreased perfusion (IVIM pseudo-diffusion coefficient [D*]) at 6 weeks after radioembolization. The combined D* and kurtosis of portal flow at baseline achieved the highest AUC (0.916) for predicting short-term response after radioembolization.

Although DWI and IVIM have wide clinical applications in HCC diagnosis, grading, and monitoring of tumor response after local-regional therapy or chemotherapy, different protocols and resultant parameters in different medical centers and scanners are the limitations of these sequences, which can result in different measurements.

## Hepatocyte-specific contrast agent

Hepatocyte-specific contrast agents include gadobenate dimeglumine (Gd-BOPTA, Multihance) and gadoxetate disodium (Gd-EOB-DTPA, Eovist, or Primovist). They are partially excreted through the kidney, partially absorbed through the organic anion transporting polypeptide 1 (OATP1B3) and excreted into the biliary system through multidrug-resistance-associated proteins (MRP2) of normal hepatocytes. They play a crucial role in the detection of small early HCC and the differentiation of benign from malignant lesions. Most HCCs appear as hypointense relative to normal background liver parenchyma in the hepatobiliary phase (HBP) because the tumor lacks normal hepatocytes, which results in a lack of hepatobiliary contrast uptake. This hypointensity characteristic correlates with the absence of OATP1B3 expression. From DNs to HCC, the expression of OATP1B3 decreases gradually during multistep hepatocarcinogenesis, particularly in the early stages of HCC. Nearly 10% of HCCs on HBP show contrast agent uptake, and the hyperintensity may be homogeneous, heterogeneous, or nodule-in-nodule in appearance. In particular, nodule-in-nodule hyperintensity, which is correlated with hepatocyte function (Yamashita et al., 2014) was speculated as a marker that can be used to predict the occurrence of HCC in the hepatocarcinogenesis process (Cannella et al., 2019). Peritumoral hyperintensity on HBP partially or wholly surrounding the lesion indicates peritumoral hyperplasia, which correlates with glutamine synthetase and OATP1B3 overexpression (Yoneda et al., 2018).

Compared to Gd-EOB-DTPA and the extracellular contrast agent (Gd-DTPA), Gd-BOPTA requires a longer scan time. This makes Gd-EOB-DTPA more widely applicable after adjusting its scanning protocol.

## Magnetic resonance elastogram/elastography (MRE)

MRE is a unique MR technique for the quantitative detection of soft tissue elasticity and structural changes. The purpose of MRE is to detect the deformation of tissues or organs under an external force and to measure the elastic mechanical parameters used in disease diagnosis. Using MRE to assess the stage of liver fibrosis with chronic liver diseases, Xiao found that MRE was a highly safe, non-invasive MR technique with high diagnostic accurary (Xiao et al., 2017). The parameter of liver stiffness can be used to evaluate the risk of HCC in patients with chronic liver disease. A study of 537 patients with hepatitis C virus infection showed that the independent factor correlated with HCC occurrence in a Cox model was an MRE value of ≥4.5 kPa (Kumada et al., 2021). The measurement of tissue stiffness can also be used to predict the occurrence of HCC in other chronic liver disorders (Figure 1B) (Motosugi et al., 2013). In an experimental study on the mechanical characterization of tumors under external compression, Pagé Gwenaël (Page et al., 2019) found that there was a significant correlation between tumor stiffness, volume change, and shear deformation. The mechanical characteristics of tumors can be improved under mechanical compression. Gordic (Gordic et al., 2017) found that tumor stiffness showed a significant negative correlation with necrosis (r = − 0.540) and a positive correlation with lesion enhancement (r = 0.514) on enhanced T1WI. Tumor stiffness is thought to be a significant biomarker for predicting the 2-year recurrence risk of HCC after tumor resection. The results showed that the mean tumor stiffness strongly correlated with tumor recurrence. They observed that the risk of HCC recurrence increased by 16.3% when the tumor stiffness was enhanced by 1-kPa (Wang et al., 2019). Another group found that liver stiffness measurements using MRE could serve as a biomarker of remnant liver regeneration after hepatectomy (Jang et al., 2017) and liver stiffness (≥4.02 kPa) in patients with HCC can be used to predict poor survival after hepatectomy (Lee et al., 2017).

This non-invasive MR technology can accurately quantify tissue stiffness and structural abnormalities. However, this technique has some limitations that restrict its widespread clinical application. It requires an additional acoustic driver and incurs additional cost. Shear wave frequency and signal acquisition may influence the measurement of tumor stiffness. In addition, specialized technicians and radiologists are needed for image reconstruction and analysis.

## T1-mapping and cT1

As a novel method, T1 relaxation time derived from T1 mapping has been primarily used for evaluating liver fibrosis; however, more recently, T1 mapping has been used for further characterization, grading (Figure 1C), and predicting the prognosis of HCC. Chen et al. (Chen et al., 2017a) investigated the T1 value in the evaluation of HCC differentiation and demonstrated that the T1 value can be used to predict the pathologic grading of HCCs with an AUC of 0.685, which is similar to that of the T1-HBP phase (T1-HBP). When combining the reduced rate of T1 values with hypointense nodules in HBP, Wang et al. (Wang et al., 2018) found a significant correlation with a higher recurrence rate after hepatectomy for HCC. Furthermore, T1 value on enhanced MRI can provide sufficient information about remnant liver function (Nakagawa et al., 2017).

Iron-corrected T1 mapping (cT1) is a novel MR biomarker that can be used to assess the increase in extracellular tissue fluid in fibroinflammatory disease with correction of iron content. cT1 showed a correlation with fibroinflammatory activity, which was confirmed by biopsy, and had a high performance in identifying patients with non-alcoholic fatty liver disease (NAFLD), nonalcoholic steatohepatitis, and related fibrosis. As the T1 value is often affected by elevated iron, it is important to eliminate this influence of elevated iron, which can be determined from the T2* maps. Using simulations with varying extracellular fluid and iron concentrations, the effects of excess iron can be removed, and a cT1 value at a normal iron level (1.3 mg/g) can be yielded (Banerjee et al., 2014). Mojtahed et al. (Mojtahed et al., 2019) established the normal cT1 reference range from 573 to 852 ms with a median of 666 ms in the low-risk group. In a clinical trial, high cT1 values (>875 ms) were strongly correlated with liver-related clinical diseases (e.g., variceal bleeding and ascites) (Pavlides et al., 2016).

The T1 relaxation time can be used to grade HCC and stage liver fibrosis. cT1 is a biomarker of liver fibrosis. It may be helpful for the TME but needs further validation for the detection of HCC metabolites.

## MR novel techniques for HCC metabolism

### MR spectroscopy

MR spectroscopy (MRS) can provide comprehensive information on the pathophysiology and metabolism of tumors. The liver is the main organ for the biological transformation and metabolism of various substances. Most of the research has focused on proton (^1^ H) and phosphorus-31 (^31^P) MRS, and only a few studies have focused on Carbon-13 (^13^C) MRS. The visible metabolites in the ^1^H MR spectra of HCC include choline, lactate, lipids, creatine, and ethanolamine-containing compounds. The major metabolites in ^31^P MRS of HCC include phosphocreatine (PCr), adenosine triphosphate (ATP), inorganic phosphate (Pi), phosphodiesters (PDEs), and phosphomonoesters (PMEs). ^13^C-labeled metabolites include 13C-labeled glucose, 13C-labeled choline, some hyperpolarized probes related to aerobic glycolysis, and pH changes.

In the process of early hepatocarcinogenesis, choline compounds, which are the most significantly enhanced substances, increase exponentially with tumor growth. It can be used as a metabolic biomarker for the quantitative and semi-quantitative evaluation of early-stage HCC. A study of a rabbit VX2 orthotopic liver cancer model showed that Cho peak area, Cho peak amplitude, and a combination of the two approaches had high sensitivity and specificity and a positive correlation with tumor diameter and volume. ^1^H-MRS can be used for the early diagnosis of HCC because choline compound levels increase exponentially. This is correlated with the increased need for choline in tumor proliferation and metabolism of the cell membrane (Liao et al., 2022). Zhang et al. calculated the choline-containing compound ratio (ΔCCC) to eliminate bio-variation. They found that, compared with benign tumors, malignant tumors showed a significant increase in the mean choline-containing compound peak area (Figure 1D). The ROC curve demonstrated high performance, with a sensitivity of 94.3% and specificity of 93.3% in diagnosing primary malignant hepatic tumors and an AUC of 0.97 (Zhang et al., 2016). Several studies have focused on ^1^H MRS for early HCC metabolic changes; the levels of choline (Cho) and lactate + triglyceride (Lac + TG) within the tumors were significantly higher than those in the peritumoral liver parenchyma and liver cirrhosis (*p* < 0.05), which also correlated with age, alkaline phosphatase, and lactate dehydrogenase. Quantification of Cho and Lac + TG can be used to predict HCC occurrence in cirrhotic livers with hepatitis, and higher levels of these metabolites may be correlated with HCC-related metabolism (Moon et al., 2020). (Wang and Li 2015) found that Cho, lipid (Lip), and Cho/Lip showed significant differences between normal liver, cirrhosis with hepatitis B, and early HCC (*p* < 0.001). Cho is associated with phospholipid metabolism in the cell membrane and is an important predictor of cell proliferation. Thus, a high concentration of Cho indicates that hepatocytes undergo continuous cell proliferation and cancerization. The increase in Cho levels in cirrhosis may be correlated with different stages of hepatocyte proliferation and the feasibility of HCC development in the future. Normal lipid levels correlate with normal liver function. Damage to liver cells inevitably leads to abnormal lipid synthesis and metabolism in patients with cirrhosis or other liver disorders. Changes in hepatic metabolite levels measured using ^1^H-MRS can help monitor the development of patients with cirrhosis.

Unlike ^1^H MRS detection of Cho, phosphorus (^31^P) MRS is used to detect energy metabolism through the levels of PCr, ATP, and Pi. Moreover, it detects the signals of the phosphomonoesters (PMEs) and phosphodiesters (PDEs). Higher PME/ATP, PME/Pi, and PME/PDE ratios indicate an increase in cell proliferation and energy metabolism, which is characteristic of tumor tissue or treatment (McKenzie et al., 2005; van der Kemp et al., 2014; Schmitz et al., 2017; Krikken et al., 2019; Meyerhoff et al., 1992; Cox et al., 1992) also reported that an elevated PME/ATP ratio, reduced ATP and Pi concentrations, and normal PDE concentrations were observed in HCC when compared to normal control livers. (Negendank 1992) analyzed hundreds of cancer studies on ^31^P and/or ^1^H MRS and found that different types of cancers showed an increase in PME, PDE, and Pi levels and a decrease in PCr level. High levels of PME, PDE, PME/Pi, and PME/Pi are the most commonly used features to differentiate normal liver from HCC. A reduction in PME and the PME/PDE ratio is a promising biomarker for predicting early response to treatment.

^13^C can be detected by MRS but has a relatively low MR sensitivity. Carbohydrates are mainly stored as glycogen in the muscles and livers of animals and humans. Several studies in animal models have shown that glycogen levels are significantly lower in hepatic tumors than in the control group. ^13^C-labeled glucose participates in glycolysis and the Krebs cycle, which reflects glucose uptake, the contributions of glycolytic pathways, oxidative phosphorylation, and provides information on oxygen consumption (Nielsen et al., 2001; Gallagher et al., 2008) found that glutamine activity increased markedly in HCC cells and that the rate of glutamine uptake and metabolism was 30-fold higher than that in normal hepatocytes. ^13^C MRS have potential clinical value in detecting small HCC with a cirrhotic background or in differentiating early HCC from DN.

Although MRS shows many advantages with respect to metabolites of HCC, the sequence requires a long acquisition time, low sensitivity in assessing smaller lesions, and additional equipment for ^31^P MRS or ^13^C MRS, which limits its wide clinical applications.

## Chemical exchange saturation transfer (CEST) MRI

As an important physiological form of energy storage, liver glycogen plays a core role in regulating blood glucose concentrations, and the glucose metabolism in HCC is essential for energy provision. Because glycogen is confined to intracellular locations, the method to measure glycogen non-invasively is of major interest in clinical applications. CEST is a novel functional MRI technique that detects chemical exchange between glycogen hydroxyl protons and tissue water. CEST MRI uses repeated marker exchange events for each acquisition, allowing for water detection and imaging of metabolites at millimolar concentrations. An experiment to measure glycogen levels using CEST in perfused mouse liver showed that glycogen levels have a strong correlation with ^13^C MRS and are more accurate than ^13^C MRS. CEST not only provides a precise and non-invasive method for the detection of liver glycogen levels and their changes but can also be used to draw glycogen distribution maps through conventional proton MRI (Miller et al., 2015).

The CEST signals are influenced by mobile macromolecules through the relayed nuclear Overhauser effect and magnetization transfer contrast from a semi-solid pool. In addition, proton exchange between bulk water and various molecules, such as amides and amines, is pH dependent. As exchange rates decrease in acidic environments, detectability decreases for amide and guanidine protons; however, OH and amine protons can be easily detected. For aerobic glycolysis in HCC, intra-tumoral/peritumoral pH_e_ differences in livers implanted with VX2 tumors were measured. The pHe within the tumor (6.8 ± 0.1) and tumor margin (6.9 ± 0.1) were significantly lower than that of normal liver (7.2 ± 0.1). Necrosis, glucose uptake, and tissue acidosis within the tumor were confirmed with histopathological markers that showed increased expression of glucose transporter 1 (GLUT-1) and lysosome-associated membrane protein 2 (LAMP-2)) (Coman et al., 2020). (Chen et al., 2017b) also validated this extracellular pH decrease in a rat hepatoma model, which was associated with tumor proliferation, staging, and response to chemotherapy. The pHe map clearly distinguished tumors and showed significantly lower values than the normal tissue. A prospective study on CEST for distinguishing hepatocellular carcinoma from hemangioma showed that CEST is translatable in clinical applications and can help detect the tumor’s pHe. The extracellular pH in the tumor region of HCC was acidic (6.66 ± 0.19) (Figure 1E), which significantly differed from the surrounding tissues of HCC (7.31 ± 0.12) and was more physiologically neutral in hemangioma (7.34 ± 0.09) (Tang et al., 2020).

CEST is a novel technique that focuses on the acidic TME in HCC. This sequence has a long scan time and limited specificity for glucose detection. Post-processing algorithms are not commercially available and are not universally available to some manufacturers.

## Blood oxygenation level-dependent MRI

Blood oxygenation level-dependent MRI (BOLD-MRI) provides a non-invasive quantitative parameter of tumor hypoxia level using the paramagnetic features of deoxyhemoglobin. Tissue T2* values can be used to reflect this change and show a negative correlation with deoxyhemoglobin levels. Based on intratumoral hypoxia and hyperperfusion of HCCs, the measurement of the blood oxygen level of HCC can be used to assess the bioactivity of HCC. Changes in blood oxygen levels in HCC can be evaluated using the T2* value. Using glucose as the simulation agent in BOLD-MRI, Yuan (Yuan et al., 2018) found that T2*values were significantly different between normal liver and background liver parenchyma of HCC and HCC centers, with a statistical decrease in the HCC center, which was correlated with the rapid growth of malignant tumors and characteristics of microvascular distribution. Tumor cells rapidly proliferate and consume oxygen, which results in hypoxia in the tumor center. Using fluorodeoxyglucose (FDG)-positron emission tomography (PET)/MRI, Hectors found significant differences in the arterial fraction and R2^∗^ value compared to HCC with background liver parenchyma (*p* < 0.032). The combination of high arterial fraction and low R2^∗^ post-O_2_ can achieve the highest diagnostic performance (AUC = 0.91) in differentiating HCC from liver background parenchyma (Hectors et al., 2018).

Bold MRI is the only non-invasive method that can be used to observe blood oxygen levels *in vivo*; however, breath-holding scans can cause motion artifacts that result in some nonidentical voxel sizes and slices.

## PET/MR in HCC metabolism

PET/MR combines MR characterizations of high soft-tissue contrast, anatomical resolution, and PET of wide metabolic information. It provides superior soft tissue contrast to accurately diagnose lesions, evaluate local extent, and perform metabolic staging. It is applicable to liver imaging and the metabolic features of HCC. Several radiotracers, including ^18^F-FDG, have been used to evaluate increases in glucose consumption, abnormalities in cell membrane metabolism, tumor proliferation, and metastasis. The avidity of ^18^F-FDG is related to the differentiation of HCC and shows high uptake in poorly differentiated HCC (Figure 1F). In contrast, choline showed high uptake in well-differentiated and moderately differentiated HCCs. Several studies have found that the combination of ^18^F-FDG and choline achieved the highest AUC for tumor detection based on tumor grading. This combination may serve as a prognostic predictor that has a poor prognosis with an FDG-PET caption. Kong et al. (Kong et al., 2017) found that the maximum standardized uptake value (SUVmax) of HCC was negatively correlated (r = −0.707) with ADC values. The SUVmax of HCC was lower than those of cholangiocarcinoma and metastases. As expected, benign lesions showed similar SUVmax and high ADC values compared to the background liver. The results showed that glucose metabolism was correlated with water molecular diffusion. ^18^F-FDG is transported *via* glucose transporters (GLUTs) in the cell membrane and accumulates in highly metabolically active cells. The expression of GLUT-1 and hexokinase type II markedly influences FDG uptake (Higashi et al., 2002). For HCC, the expression of GLUT-1 is weak but increases the expression of hexokinase type II (Lee et al., 2005). SUV is correlated with tumor proliferation and can help characterize malignancy (Higashi et al., 2000). In contrast, hypercellular areas with a high proliferative index have a higher cellular density and more restricted water molecular diffusion, resulting in lower ADC in malignant tumors. (Parsai et al., 2019) found that PET/MR for characterizing malignant tumor achieved the highest performance compared with the MRI or PET alone, and the sensitivity, specificity, and accuracy were 91.9%, 97.4%, and 94.7%, respectively. For differentiating malignant and benign lesions, PET/MR combined with HBP was superior to PET and MRI without HBP (*p* < 0.001). It enhances the diagnostic performance and reliability in accurately evaluating lesions (Kirchner, Sawicki et al., 2017). However, the potential value of PET/MR imaging for HCC may be liver-specific or malignant-tumor-related radiotracers (i.e., choline), such as a prostate-specific membrane antigen for detecting prostate cancer or CXC chemokine receptor type 4-based radiotracers for solid malignant tumors or hematologic malignancies, which can provide unique information about tumor pathophysiology.

PET/MR provides metabolic information and prognostic factors for HCC; however, its high cost, long acquisition time, and weak FDG activity within well-differentiated HCC make it less applicable for HCC detection.

Table 1 summarizes the different techniques discussed in this review, their clinical applications, advantages, and limitations.

**TABLE 1:** Comparison of techniques used for characterization of hepatocellular carcinoma (HCC) metabolism.

Technique	Sequence parameters	Biological changes	Clinical applications	Advantages	Limitations
DWI and IVIM	ADC, D, D*, f	Microscopic changes in water mobility HCC cellularity, and micro-perfusion value	Tumor detection, diagnosis, grading and staging, and evaluation of early response of local regional therapy and chemotherapy	Well-established conventional MR sequence, short acquisition time, and high contrast resolution	Different protocols and resultant parameters in different medical centers and scanners
Hepatocyte-specific contrast agent (Gd-BOPTA and Gd-EOB-DTPA)	Tumor enhancement type, hypointense in HBP	Hyper-vascular tumor, lack of OATP1B3 of HCC	Tumor early detection and characterization, and evaluation of response after therapy	Well-established Conventional MR sequences Combination features of extracellular contrast agents and HBP, detection and characterization of HCC significantly improved	Longer scan time with Gd-BOPTA-enhanced MRI compared with Gd-EOB-DTPA and extracellular contrast agents
MRE	Tumor mean stiffness	tumor elasticity and structural changes	Tumor detection in the patients with chronic liver disease and prediction of early recurrence after surgery	Non-invasive, accurate, and quantitative measurement of soft tissue elasticity and structural abnormality	Need additional external driver, shear wave frequency, signal acquisition, software influence the parameters, and requires specialized technician and radiologist
T1-mapping and cT1	T1 relaxation time Iron-corrected T1	Extracellular fluid and fibroinflammatory changes	Staging liver fibrosis and prediction fibroinflammatory disease, evaluation the differentiation of HCC, and evaluation of liver or remnant liver function	Non-invasive quantitative method for staging liver fibrosis and superiority for detection of fibroinflammatory changes	Need further validation for HCC metabolism
MRS	Cho, Lac + TG, Lip PCr, ATP, Pi, PMEs, PDEs ^13^C-labeled glucose	Increased need for Cho in tumor proliferation and enhanced metabolism of energy in HCC	Metabolites detection of HCC and prediction of response to treatment	Acceptable accuracy and diagnostic performance of tumor metabolism and simultaneous measurement of pHe	Long acquisition time, low sensitivity in assessing smaller lesions, and requires additional instrument for^31^P or^13^C MRS
CEST	pHe	Abnormality of glucose metabolism of HCC Extracellular acid tumor microenvironment	PHe inside HCC and detection of liver glycogen levels Differentiation of HCC from benign tumor	Non-invasive method for measuring extracellular pH (pHe)	Long scan time, limited specificity for detecting glucose, and data postprocessing
BOLD-MRI	R2*	Change of deoxyhemoglobin concentration within HCC	Tumor hypoxia microenvironment and evaluation of early changes after chemotherapy	Non-invasive methods for evaluating tumor pathophysiologic state and the level of hypoxia in tumors	Breath-holds cause many motion artifacts, and voxel size and number of slices was not identical
PET/MR	SUV and multiparametric MR parameters	Abnormality of tumor biology and metabolism	Providing metabolism information of HCC, prognostic factors, and extrahepatic staging; evaluating treatment response; and predicting HCC recurrence after surgery or transplant	Improved tissue contrast resolution, lack of ionizing radiation, and quantitative measurements of tissue biology	Higher cost, long acquisition time, and weak FDG activity within some HCC

Abbreviations: DWI, diffusion-weighted imaging; IVIM, intravoxel incoherent motion; ADC, apparent diffusion coefficient; D, diffusion coefficient; D*, pseudo-diffusion coefficient; f, perfusion fraction; HBP, hepatobiliary phase; OATP1, organic anion transporting polypeptide one; MRE, magnetic resonance elastography; cT1, iron-corrected T1 relaxation time; MRS, magnetic resonance spectroscopy; Cho, choline; Lac + TG, lactate and triglyceride; Lip, lipid; PCr, phosphocreatine; ATP, adenosine triphosphate; Pi, inorganic phosphate; PMEs, phosphomonoesters; PDEs, phosphodiesters; CEST, chemical exchange saturation transfer; BOLD-MRI, blood oxygenation level-dependent MRI; PET/MR, positron emission tomography/magnetic resonance; SUV, standardized uptake value.

In conclusion, altered cellular metabolism and a complex TME are major characteristics of HCC. Multiparametric MRI and PET/MRI provide accurate information about tumor structural and functional changes, metabolite detection produced by malignant tumor cells, and the interaction between malignant tumor cells and the TME. This may further facilitate and improve individual treatments for patients with HCC.
